# Non-epithelial Circulating Tumor Cells Enhance Disease Progression in High-risk Prostate Cancer through EMT and COL1A1 Expression

**DOI:** 10.7150/ijms.107703

**Published:** 2025-02-28

**Authors:** Yiyuan Li, Ruji Wu, Hua Wang, Meinong Zhong, Yunhao Qing, Shuo Lu, Zixiao Zhang, Tan Ma, Jieheng Luo, Hengjun Xiao, Jianguang Qiu, Ke Li

**Affiliations:** 1 Department of Urology, The Sixth Affiliated Hospital, Sun Yat-Sen University, Guangzhou, China.; 2 Department of Urology, SSL Central Hospital of Dongguan City, Dongguan, China.; 3 Department of Urology, The Third Affiliated Hospital, Sun Yat-sen University, Guangzhou, China.; 4 Biomedical Innovation Center, The Sixth Affiliated Hospital, Sun Yat-sen University, Guangzhou, China.

**Keywords:** prostate cancer, circulating tumor cells, non-epithelial CTCs, prognosis, COL1A1

## Abstract

**Introduction:** Circulating tumor cells (CTCs) are important prognostic indicators for malignancies. However, a reliable positive/negative cutoff value of non-epithelial (NE^+^: hybrid and mesenchymal) CTCs phenotype in prostate cancer (PCa) patients has not been established. Here, we aimed to determine the cutoff value and the prognostic value of NE^+^ CTCs in high-risk prostate cancer (HRPC) patients after radical prostatectomy (RP).

**Methods:** The cutoff value of NE^+^ CTCs was established in spiking experiments, and CTCs were detected in 208 HRPC patients using the CanPatrol^TM^ platform. The expression and function of COL1A1 in PCa were examined via qRT-PCR, Western blot, wound healing assay, Transwell assay, and immunohistochemistry (IHC).

**Results:** The cutoff value of NE^+^ CTCs was determined to be 45% by spiking experiments. In 208 HRPC patients, the NE^+^ CTCs positive group had higher prostate-specific antigen (PSA) levels, more advanced pathological tumor stage, and lymph node stage (*P* < 0.001, *P* = 0.002 and 0.002, respectively). Besides, patients with NE^+^ CTCs ≥ 45% had a shorter median progression-free survival (PFS) than those with NE^+^ CTCs < 45% (44.5 vs. 51.0 months, hazard ratio = 3.31, *P* < 0.05). Moreover, we identified that COL1A1 was associated with a high proportion of NE^+^ CTCs in HRPC patients via an EMT mechanism.

**Conclusion:** Our findings suggest that NE^+^ CTCs represent a reliable prognostic indicator for HRPC patients and that targeting COL1A1 may prevent the formation of NE^+^ CTCs.

## Introduction

Prostate cancer (PCa) is the second leading cause of cancer death in males, and the prognosis varies significantly across PCa patients^1^, ^2^. Therefore, risk stratification for prognosis is crucial in PCa management. However, conventional prognostic markers such as prostate-specific antigen (PSA), Gleason score, and tumor stage cannot reliably predict survival outcomes^3^, ^4^. Circulating tumor cells (CTCs), detected by liquid biopsy techniques, provide noninvasive prognostic information for a range of malignancies^5^, ^6^. Studies have reported that the molecular phenotype of CTCs holds substantial prognostic value, and classification based on epithelial-mesenchymal transition (EMT) is one of the major clinical methods for categorizing CTCs^7^, ^8^. The transition from epithelial to mesenchymal CTCs is accompanied by increased metastatic potential and a worse prognosis^9^. However, reports revealed that metastasis does not necessitate a complete EMT^10^, ^11^. The hybrid CTCs, which underwent partial EMT, had a stronger capacity to develop lung metastases than mesenchymal CTCs^12^. Moreover, the dynamic fluctuation between EMT and mesenchymal-epithelial transition (MET) makes it more challenging to predict outcomes using a single subtype of CTCs^13^. Therefore, novel strategies are urgently required to improve the efficiency of EMT-based molecular characterization of CTCs.

Here, we sought to determine the cutoff value for non-epithelial (NE^+^: hybrid and mesenchymal) CTCs in PCa by spiking experiments and to assess the prognosis value of NE^+^ CTCs in patients with high-risk PCa (HRPC) following radical prostatectomy (RP). Additionally, we performed bioinformatics analysis and experimental validation to investigate the genes contributing to the elevated proportion of NE^+^ CTC in HRPC patients.

## Materials and Methods

### Patients and blood samples collection

A total of 208 patients with histologically diagnosed prostate adenocarcinoma were enrolled at The Third Affiliated Hospital of Sun Yat-sen University from June 2016 until June 2018 and followed up from June 2016 to June 2022. Patients were eligible for inclusion if they were stratified into a high-risk group according to the D'Amico classification system^14^ (prostate-specific antigen (PSA) ≥ 20 ng/mL, clinical T stage ≥ cT2c, or Gleason score ≥ 8) and had a life expectancy of longer than 5 years. Exclusion criteria included evidence of distant metastasis on imaging studies, previous androgen deprivation therapy (ADT), prostatectomy, radiotherapy, chemotherapy and other treatments, and malignancy within the previous 5 years. All patients underwent radical prostatectomy (RP), and 5 mL of peripheral blood was drawn before the surgery. Patients received adjuvant ADT (goserelin acetate 3.6 mg subcutaneously in the upper abdominal wall every 28 days) and were followed up every month after RP. Disease progression was defined as two sequential increasing PSA values > 0.2 ng/mL or any radiological progression.

### Circulating tumor cells (CTCs) isolation and classification

The CanPatrol^TM^ platform was employed to isolate CTCs as previously described^15^. Epithelial and mesenchymal markers were detected on CTCs in all prostate cancer (PCa) patients. A tri-color RNA *in situ* hybridization (RNA-ISH) assay based on branched DNA (bDNA) signal amplification technology was used to classify CTCs, with capture probes shown in the **Supplementary Materials**.

### Cancer cell lines and cell spiking experiments

Based on expression level and tumor histology, we selected the metastatic (PC3, DU145, LNCaP) and local (22Rv1) prostate cancer cell lines to establish cutoff values. All cell lines were obtained from the American Type Culture Collection (ATCC) and grown in RPMI1640 medium (GIBCO, USA) containing 10% fetal bovine serum (GIBCO, USA). Details of cell spiking experiments are provided in the **Supplementary Materials**. The capture probes specific for epithelial/ mesenchymal biomarkers (epithelial biomarkers: EpCAM and CK8/18/19; mesenchymal biomarkers: vimentin and twist) were used to classify CTCs. Non-epithelial (NE^+^) CTCs were defined as hybrid and mesenchymal CTCs.

### Data source

The present study obtained the clinical data and corresponding raw transcriptome count data for PCa patients from the TCGA database (https://portal.gdc.cancer.gov/). Besides, the GSE32269, GSE38241, and GSE60329 datasets were acquired from the Gene Expression Omnibus (GEO) database using the keyword “prostate cancer” in NCBI (https://www.ncbi.nlm.nih.gov/). The EMT-related gene set (HALLMARK_EPITHELIAL_MESENCHYMAL_TRANSITION) was acquired from the MSigDB database (https://www.gsea-msigdb.org/gsea/index.jsp). The **Supplementary Materials and Table S1** contain more information about the above databases.

### Analysis of differentially expressed genes (DEGs) between PCa and normal samples

Firstly, we conducted background correction and normalization on the publicly available microarray data. Next, using the “limma” R package^16^, we performed differential expression analysis and identified the DEGs between PCa tissues and normal tissues in both TCGA, GSE60329, GSE32269, and GSE38241 datasets. The *P* values were adjusted for multiple testing corrections by false discovery rate (FDR)^17^. DEGs were recruited with |log2FC| value > 1 and FDR < 0.05.

### Quantitative reverse transcription polymerase chain reaction (qRT-PCR)

Total RNA was extracted using the TRIzol reagent (Invitrogen, Grand Island, NY, United States), and reverse transcription was carried out using the HiScript III RT SuperMix for qPCR (Vazyme, China). SYBR Green Pro Taq HS (Agbio, China) was used for qRT-PCR on a LightCycler^®^ 480 System (Roche). The PCR protocol included 45 cycles at 95°C for 5 minutes (min), then 95°C for 10 seconds (s), 60°C for 10 s, and 72°C for 10 s. Based on the 2^-ΔΔCt^ method, the relative mRNA levels were normalized against GAPDH. The sequences of the qRT-PCR primers are listed in **Table S2**.

### Western blot

Proteins (30-50 μg) extracted from cells were diluted 1:4 (v/v) in loading buffer (Beyotime, China), heated at 95 °C for 10 min, and loaded onto 10% gels (1.5 × 10 wells; EpiZyme, China). The samples were electrophoresed at 180 V for 50 min and transferred to PVDF membranes (Millipore, Billerica, MA, USA) at 100 V for 90 min. The membranes were blocked with 5% nonfat milk for 1 hour (h) and incubated with primary antibodies overnight at 4 °C, followed by incubation with secondary antibodies for 60 min at room temperature. The membrane exposure was conducted using an ECL kit (Biosharp, China) and ChemiDoc Imaging System. The primary antibodies used were as follows: COL1A1 (A16891) was from ABclonal, (E-Cadherin (#3195), N-Cadherin (#13116), Vimentin (#5741) and GAPDH (#97166) were from Cell Signaling Technology.

### Cell viability assay

Cell viability was measured using the Cell Counting Kit-8 (CCK-8, ApexBio, MA, USA) according to the manufacturer's instructions. Briefly, PC3 and 22Rv1 cells were seeded at a density of 8 × 10^3 cells/well and 1 × 10^4 cells/well in 96-well culture plates, respectively. The cells were then transfected with siRNA oligonucleotides targeting human COL1A1 and siRNA control after reaching 50-60% confluence. Finally, after 24 h, 48 h, and 72 h of transfection, 10 μL of CCK8 reagent was added to each well, and the culture plates were incubated at 37°C for 2 h. The absorbance at 450 nm was measured using a microplate reader (Thermo Fisher Scientific, USA).

### Wound healing assay

PC3 and 22Rv1 cells were seeded and grown to 100% confluence in six-well plates. Wounds were created in the center of the cell monolayer and observed using a phase-contrast microscope at the indicated time point (PC3: 24 h, 22Rv1: 72 h). Four distinct positions were selected to measure the wound's width. The ratio between the final and initial real wound widths was used to calculate relative wound width.

### Transwell assays

Cell migration and invasion capabilities were investigated using 24-well Transwell inserts with 8 μm pores (Falcon, Corning, NY, United States). Briefly, transfected cells (PC3: 3 × 6 10^4 cells, 22Rv1: 5 × 10^4 cells) were suspended in 200 μL of serum-free medium and seeded into the upper chamber of a Transwell insert, which was precoated without (migration) or with (invasion) Matrigel (Corning, United States). The lower chamber was filled with a medium containing 10% FBS as a chemoattractant. After incubation for the appropriate time (PC3: 24 h, 22Rv1: 72 h), cells in the upper chamber were gently removed, and migrated or invaded cells on the lower side of the Transwell membrane were fixed, stained and observed by microscopy (Olympus). Five random visual fields were chosen for each chamber, and each experiment was repeated independently at least three times.

### COL1A1 knockdown by siRNA

The siRNA oligonucleotides for human COL1A1 and siRNA control were purchased from Ribobio (Guangzhou, China). PC3 and 22Rv1 cells were transfected using jetPRIME^®^ (Polyplus-transfection S.A, Illkirch, France). After 24 h, the transfection medium was replaced with a fresh culture medium. Validation of mRNA and protein by qRT-PCR and Western blot was performed 48h and 72 h after transfection, respectively. The sequences of the siRNA are listed in **Table S3**.

### Tumor tissue immunohistochemistry (IHC)

Semi-quantitative IHC was performed by multiplying the level of staining intensity, as previously described^18^. The COL1A1 antibody was purchased from ABclonal (A16891). Details on the assessment of COL1A1 staining are shown in **Supplementary Materials**.

### Statistical analysis

Clinicopathological characteristics were compared between groups using the chi-square test. Correlations between tissue COL1A1 expression levels and NE^+^ CTCs percentages were examined by Kruskal-Wallis analysis and Mann-Whitney U test. Gene expression data from the TCGA and GTEx databases were analyzed using Student's t-test. Progression-free survival (PFS) was estimated using a Cox regression model, and the assumption of proportionality was verified by examination of Kaplan-Meier curves. Statistical analyses were performed using SPSS version 27.0 software (IBM, USA). Scatter plots and histograms were generated using GraphPad Prism 9. All statistical tests were 2-sided, and *P* < 0.05 were considered statistically significant.

## Results

### Establishment of the non-epithelial (NE^+^) circulating tumor cells (CTCs) percentage cutoff value in prostate cancer (PCa)

**Figure 1:**
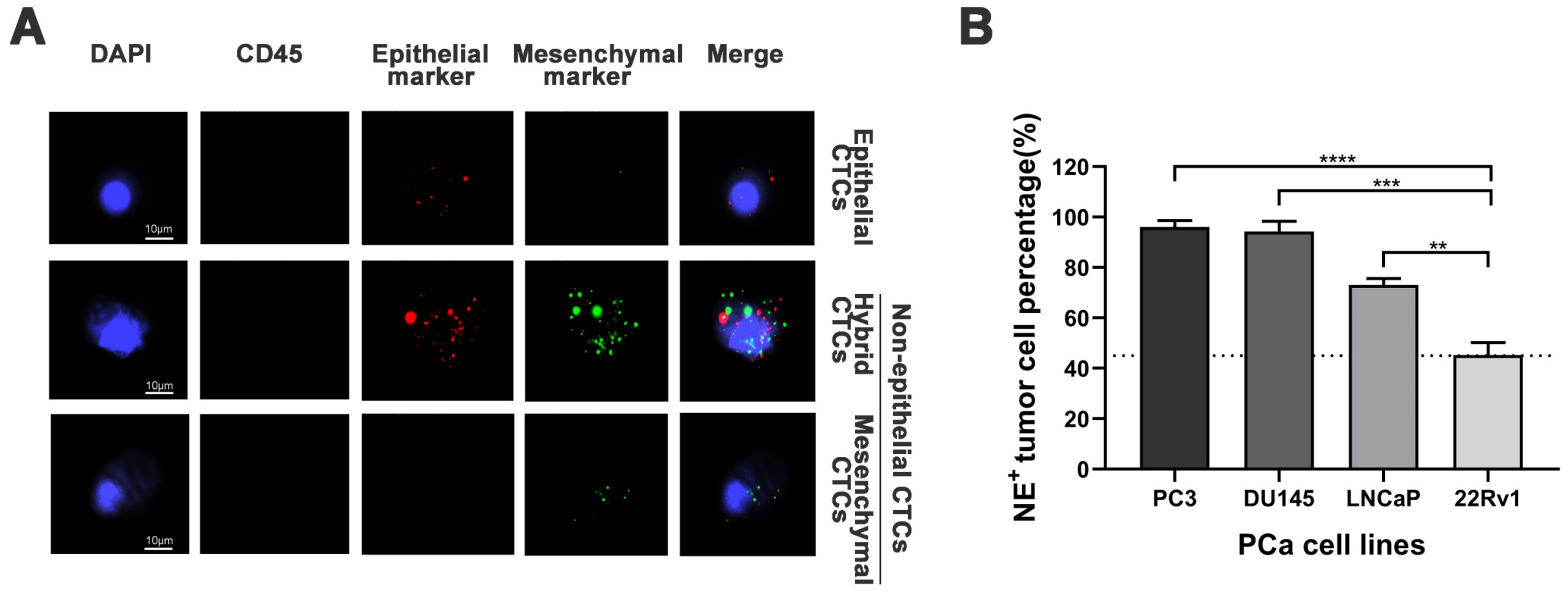
** Establishment of the cutoff value for non-epithelial (NE^+^) circulating tumor cells (CTCs) percentage. A)** Representative tri-color RNA *in situ* hybridization (RNA-ISH) images illustrating different subtypes of CTCs (NE^+^ CTCs defined as hybrid/mesenchymal CTCs). Probe targets: Red: epithelial markers (EpCAM and CK8/18/19). Green: mesenchymal markers (vimentin/twist). Blue: DAPI (nuclei staining). Scale bar = 5 µm.** B)** NE^+^ tumor cells percentage across four prostate cancer cell lines in spiking experiments. The cutoff value (dashed line) was defined as the lowest observed percentage (22Rv1, 45%). Data represent mean ± standard deviation (SD) of three independent experiments. **P < 0.01, ***P < 0.001, ****P < 0.0001, according to Student's *t*-test.

Representative images of epithelial/mesenchymal markers in CTCs were displayed in **Figure 1A**, while the results of the spiking experiments were presented in **Figure 1B**. The experiments revealed the highest percentage of NE^+^ CTCs in the PC3 cell line (96.1%) and the lowest percentage of NE^+^ CTCs in the 22Rv1 cell line (45.2%). The cutoff value for NE^+^ CTCs was defined as the lowest observed percentage, which was 45%. Thus, Patients with a NE^+^ CTCs percentage ≥ 45% and < 45% were categorized into NE^+^ CTCs positive group and NE^+^ CTCs negative groups, respectively.

### NE^+^ CTCs percentage stratifies high-risk prostate cancer (HRPC) patients by clinicopathological features

CTCs were detected in 187 of 208 HRPC patients (89.9%), with a median total CTCs (t-CTCs) count of 5 / 5 mL (range: 0-78 / 5 mL). Patients were categorized into a t-CTCs positive group (≥ 5 / 5 mL) and a negative group (< 5 / 5 mL) based on the t-CTCs count, as previously described^19^. Prostate-specific antigen (PSA), biopsy Gleason score (biopsy GS), clinical lymph node stage (cN), pathological GS (pGS), pathological lymph node stage (pN), and surgical margins were significantly associated with t-CTCs count (*P* = 0.008, 0.009, 0.005, 0.003, 0.002 and 0.040, respectively, **Table 1**).

Additionally, NE^+^ CTCs were observed in 175 of 208 patients (84.1%), and the median NE^+^ CTCs count was 4 / 5 mL (range: 0-42 / 5 mL). Using the established cutoff value of 45%, 168 patients (80.8%) were classified as NE^+^ CTCs positive group (≥ 45%), while 40 patients (19.2%) were in the negative group (< 45%). The NE^+^ CTCs percentage was significantly associated with PSA, cN, pathological tumor stage (pT), pN, and surgical margins (*P* < 0.001, *P* = 0.016, 0.002, 0.002, and 0.016, respectively, **Table 1**).

### High NE^+^ CTCs percentage predicts worse postoperative outcomes in HRPC patients

**Figure 2:**
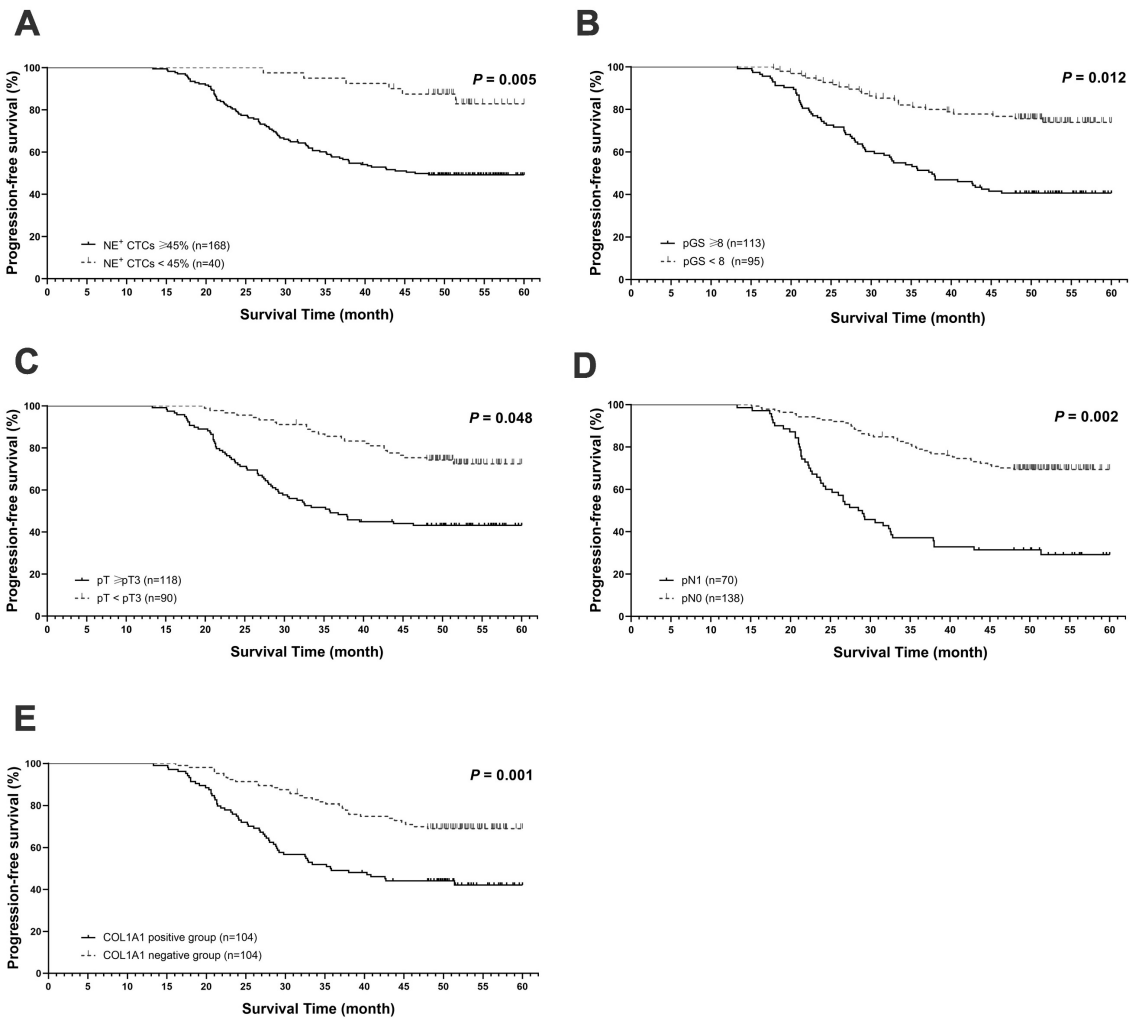
** Prognostic value of NE^+^ CTCs percentage in postoperative high-risk prostate cancer (HRPC) patients.** Progression free-survival (PFS) based on **A)** NE^+^ CTCs percentage (≥ 45% vs. < 45%). **B)** Pathological Gleason score (pGS, ≥ 8 vs. < 8). **C)** Pathological tumor stage (pT, ≥ pT3 vs. < pT3). **D)** Pathological lymph node stage (pN, pN1 vs. pN0). **E)** COL1A1 expression (positive vs. negative). PFS was estimated using a Cox regression model, and the assumption of proportionality was verified by examination of Kaplan-Meier curves.

During a median follow-up period of 50.5 months (range, 21.0-60.0 months), 91 patients (43.8%) experienced disease progression. The median PFS for the t-CTCs positive and the t-CTCs negative groups were 48.0 months and 49.2 months, respectively. While in NE^+^ CTCs positive group and NE^+^ CTCs negative group, the PFS were 44.5 months and 51.0 months, respectively. In univariate Cox regression analysis, both NE^+^ CTCs percentage and t-CTCs count were correlated with poorer survival (*P* = 0.039 and *P* < 0.001). However, multivariate analysis revealed that the NE^+^ CTCs percentage, but not the t-CTCs count, were an independent predictor for progression (hazard ratio [HR] = 3.31; 95% confidence interval [CI]: 1.423-7.702, *P* = 0.005, **Table 2 and Figure 2A**). Additionally, pGS, pT, and pN were independent prognostic factors for progression (**Table 2 and Figure 2B-D**).

### Bioinformatic analysis of epithelial-mesenchymal transition (EMT)-related genes in PCa

To identify the genes that may drive the formation of CTCs, particularly NE^+^ CTCs in HRPC patients, we applied an integrated analysis of EMT-related genes in PCa.

**Figure 3:**
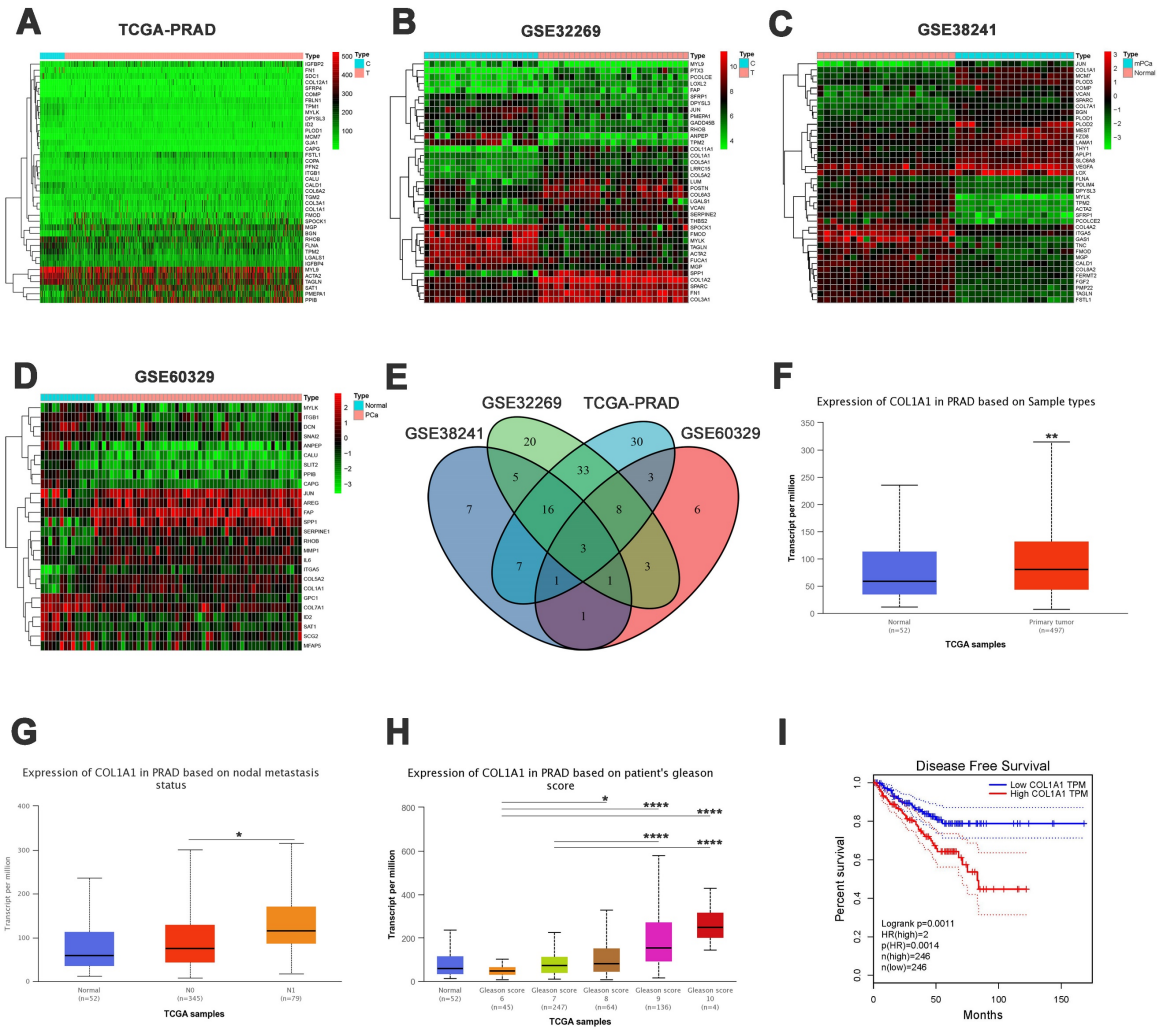
** Bioinformatic analysis of epithelial-mesenchymal transition (EMT)-related genes in PCa. A-D)** Heatmaps of differentially expressed genes (DEGs) in TCGA-PRAD, GSE32269, GSE38241, and GSE60329 datasets.** E)** Venn diagram of the overlapping DEGs (COL1A1, MYLK, and ITGB1). **F)** COL1A1 expression in TCGA-PRAD samples between PCa and normal tissue.** G)** COL1A1 expression in TCGA-PRAD samples among nodal metastasis subgroups. **H)** COL1A1 expression in TCGA-PRAD samples based on Gleason score. **I)** Disease-free survival (DFS) by COL1A1 expression. Data represent mean ± SD. *P < 0.05, **P < 0.01, ***P < 0.001, ****P < 0.0001, according to Student's t-test.

Initially, we obtained the EMT-related gene set from the MSigDB database. Using the “limma” package, we identified EMT-related differentially expressed genes (DEGs) between PCa and normal prostate samples in the TCGA dataset. The DEGs were presented in a heatmap (**Figure 3A**) and a volcano plot (**Figure S1A**). The same analysis was conducted on the GSE32269, GSE38241, and GSE60329 datasets. The corresponding heatmaps and volcano plots are presented in **Figure 3B-D and Figure S1B-D**. A Venn diagram revealed three overlapping genes among the different groups: COL1A1, MYLK, and ITGB1 (**Figure 3E**).

Previous studies have suggested that COL1A1 may predict the prognosis of prostate cancer^20^, ^21^. Therefore, we focused on the expression and function of COL1A1, particularly examining its regulatory role in EMT progression. First, we conducted an expression and clinical correlation analysis using the UALCAN database, which revealed significantly higher transcription levels of COL1A1 in PCa tissue than in normal tissue (**Figure 3F**). Besides, compared to patients without nodal metastasis, those with nodal metastasis had higher transcription levels of COL1A1 (**Figure 3G**). Similarly, there was a significant association between high levels of COL1A1 and high Gleason scores (**Figure 3H**). The above results indicate a potential promoting role of COL1A1 signaling in PCa metastasis.

Moreover, to evaluate the impact of COL1A1 expression on the prognosis of PCa patients, we used the GEPIA2 database to assess the correlation between COL1A1 expression and clinical outcomes. The disease-free survival (DFS) curve shown in **Figure 3I** illustrated that those patients with higher transcription levels of COL1A1 had significantly poorer DFS (*P* = 0.0011). However, an overall survival (OS) analysis implied that COL1A1 expression did not affect the OS of PCa patients (*P* = 0.11, **Figure S1E**). In summary, COL1A1 may be a negative prognostic factor for prostate cancer.

### COL1A1 facilitates tumor progression by activating EMT signaling pathway in PCa cells

**Figure 4:**
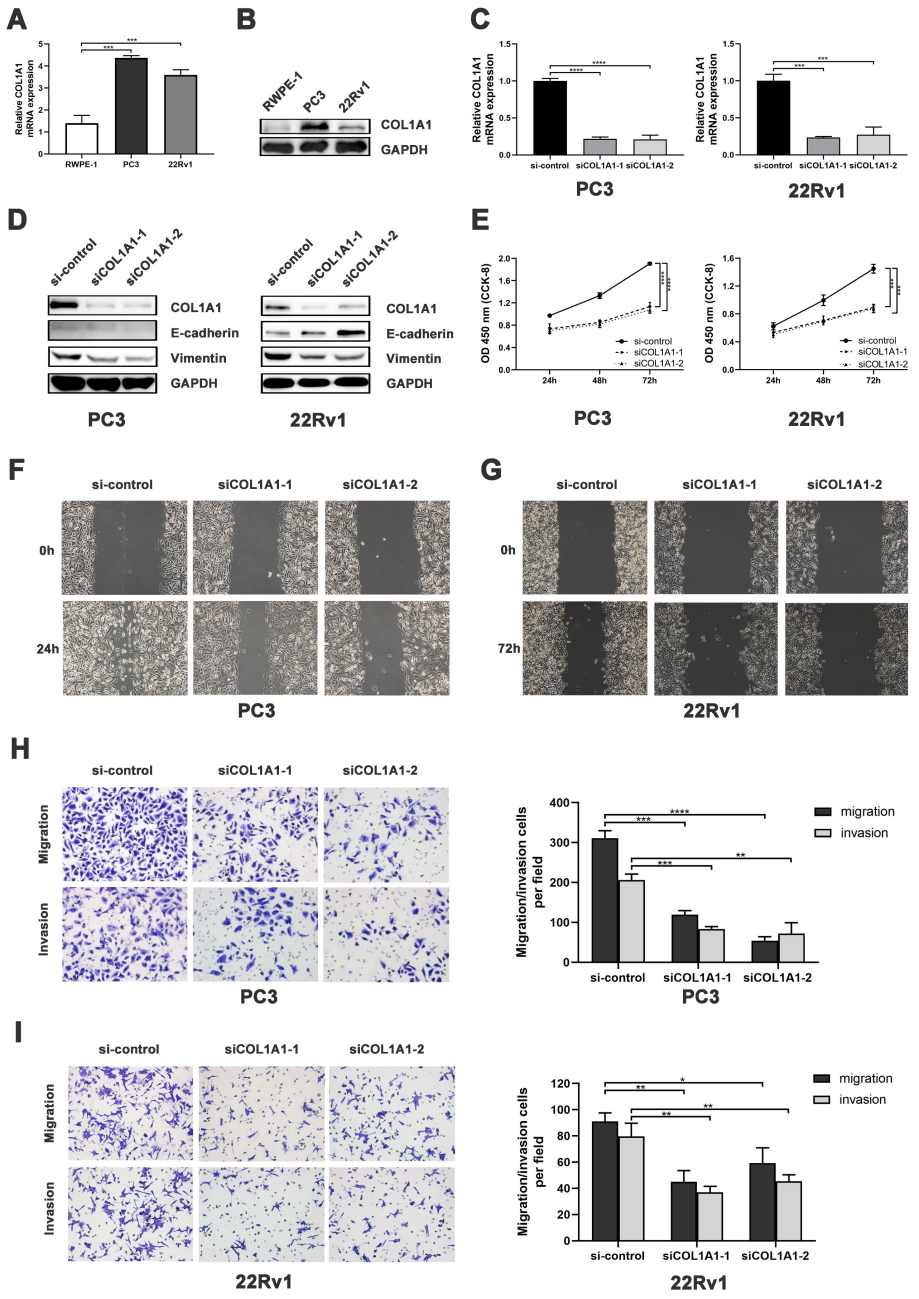
** COL1A1 facilitates PCa progression by EMT activation. A-B)** COL1A1 mRNA/protein expression in PCa cell lines (PC3/22Rv1) and human prostate epithelial cell line (RWPE-1).** C)** qRT-PCR validation of COL1A1 siRNA knockdown. **D)** Western blot showing EMT protein changes after COL1A1 knockdown.** E)** CCK-8 assay showing effects of COL1A1 knockdown on cell viability and proliferation. **F-G)** Wound healing assay showing effects of COL1A1 knockdown on the migration. **H-I)** Transwell cell assay showing effects of COL1A1 knockdown on migration and invasion. Data represent mean ± SD of three independent experiments. *P < 0.05, **P < 0.01, ***P < 0.001, ****P < 0.0001, according to Student's *t*-test.

We first measured the COL1A1 expression in PCa cell lines. Quantitative Real-Time quantitative PCR (qRT-PCR) revealed that the mRNA level of COL1A1 in PC3 and 22Rv1 cells was significantly higher than in RWPE-1 cells (**Figure 4A**). Consistently, Western blot analysis showed that the protein expression of COL1A1 in PC3 and 22Rv1 cells was markedly higher than in RWPE-1 cells (**Figure 4B**).

Subsequently, siRNA was used to down-regulated COL1A1 expression in PC3 and 22Rv1. As shown in **Figure 4C-D**, both the mRNA and protein levels of COL1A1 were effectively decreased in PC3 and 22Rv1 cells after siRNA transfection. After that, Cell Counting Kit-8 (CCK-8) assays revealed that the knockdown of COL1A1 significantly reduced cell viability in both PC3 and 22Rv1 cells (**Figure 4E**). In addition, the wound healing assay and the Transwell cell assay showed that the knockdown of COL1A1 significantly repressed cell migration and invasion capacity (**Figure 4F-I**).

Next, we investigated the alteration of EMT markers E-cadherin and vimentin after COL1A1 silencing. Western blot showed that knockdown of COL1A1 downregulated the expression of COL1A1 and vimentin protein in both PC3 and 22Rv1. Additionally, COL1A1 knockdown upregulated the expression of E-cadherin protein in 22Rv1 (**Figure 4D**). These results suggest that COL1A1 plays a critical role in EMT in PCa.

Overall, the above findings suggest that COL1A1 may promote the migration and invasion of PCa through EMT.

### Tissue COL1A1 expression associates with NE^+^ CTCs percentage in HRPC patients

**Figure 5:**
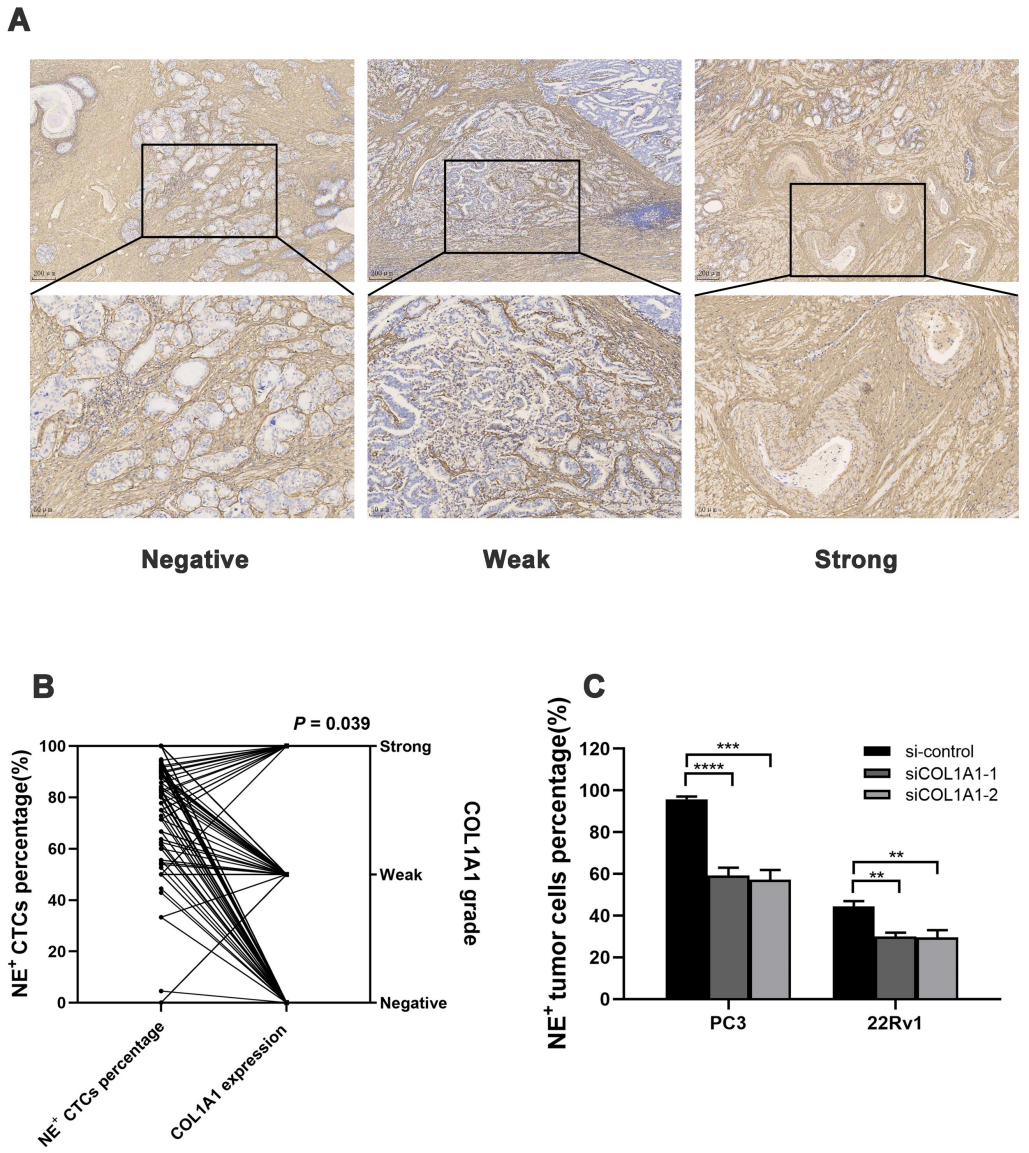
** Association between tissue COL1A1 expression and NE^+^ CTCs percentage in HRPC. A)** Representative image of COL1A1 immunohistochemistry (IHC) staining in HRPC surgical specimens.** B)** NE^+^ CTCs percentage across different COL1A1 staining grade subgroups. *P*-value was estimated by Kruskal-Wallis analysis. **C)** NE^+^ tumor cell percentage reduction after COL1A1 knockdown by spiking experiments. Data represent mean ± SD of three independent experiments. **P < 0.01, ***P < 0.001, ****P < 0.0001, according to Student's *t*-test.

Next, the COL1A1 expression in surgical specimens from 208 HRPC patients was evaluated by immunohistochemistry (IHC), and 104 patients had positive expressions (**Figure 5A**). COL1A1 expression in tissue was associated with pN (*P* = 0.019). Besides, COL1A1 was an independent predictor for postoperative progression in HRPC patients (hazard ratio [HR] = 2.17; 95% confidence interval [CI]: 1.398 - 3.377, *P* = 0.001, **Figure 2E and Table S4**). Moreover, we observed positive COL1A1 staining in 96 cases in the NE^+^ CTCs positive group and only 8 cases in the negative group. Statistical analysis revealed a significant difference in the percentage of NE^+^ CTCs across different COL1A1 staining grades (*P* = 0.039, **Figure 5B**), and pairwise comparison showed the percentage of NE^+^ CTCs was significantly higher in patients with strong COL1A1 positivity than in those with COL1A1 negativity (*P* = 0.020). Furthermore, spiking experiments were conducted to investigate the impact of COL1A1 knockdown on NE^+^ tumor cells. The results demonstrated a significant decrease in the percentage of NE^+^ PC3 and 22Rv1 cells (**Figure 5C**).

Taken together, these findings revealed that COL1A1 is up-regulated in PCa and positively related to the percentage of NE^+^ CTCs in HRPC patients, potentially mediated by an EMT regulatory mechanism.

## Discussion

Currently, in prostate cancer (PCa), it remains challenging to determine an objective cutoff value for non-epithelial (NE^+^) circulating tumor cells (CTCs). Most studies employed the count of CTCs as a criterion, for example, total CTCs ≥ 5 cells or mesenchymal CTCs ≥ 2 cells^19^, ^22^. Intriguingly, mesenchymal CTCs percentages were explored in several studies^7^, ^23^, ^24^, demonstrating our strategy's feasibility. Inspired by the previous literature^25^, we conducted spiking experiments with four heterogeneous PCa cell lines and evaluated the proportion of NE^+^ tumor cells. The spiking experiments showed that the NE^+^ percentage in 22Rv1 cells was relatively low. Therefore, the cutoff value for NE^+^ CTCs was determined to be the NE^+^ tumor cells percentage in 22Rv1 cells.

Subsequently, our study validated the cutoff value of NE^+^ CTCs in patients. We found that NE^+^ CTCs were an independent prognostic factor for progression-free survival (PFS) in high-risk prostate cancer (HRPC) patients. Additionally, NE^+^ CTCs were significantly correlated with prostate-specific antigen (PSA), pathological tumor stage (pT), pathological node stage (pN), and surgical margins. Mesenchymal CTCs were reported to be more aggressive and unstable than epithelial CTCs^26^. However, hybrid CTCs, the immediate type, most effectively predicted metastasis in pancreatic ductal adenocarcinoma and intrahepatic metastasis in hepatic cell carcinoma^27^, ^28^. Besides, a reverse conversion mesenchymal-epithelial transition (MET), which plays a crucial part in the metastatic PCa cell colonization and growth in the proximity of the bone microenvironment^29^, ^30^, may change the ratios of mesenchymal and hybrid CTCs. Finally, given the minimal number of CTCs, further distinctions between the hybrid and mesenchymal subtypes might not yield significant benefits. Combining hybrid and mesenchymal subtypes into NE^+^ CTCs may be a more effective tactic to reflect the tendency of CTCs for metastasis.

To investigate which genes are the cause of the high percentage of NE^+^ CTCs in HRPC patients, we performed an integrated analysis. Among the epithelial-mesenchymal transition (EMT)-related genes, we identified COL1A1, which has been linked to metastasis in PCa^21^, ^31^. Online clinical data revealed that COL1A1 was substantially expressed in PCa and connected to metastasis and DFS. In the database clinical data, COL1A1 was highly expressed in PCa and associated with metastasis and disease-free survival (DFS). Further *in vitro* experiments showed that the knockdown of COL1A1 significantly inhibited the proliferation, invasion, and migration of PCa. Critically, COL1A1 knockdown suppressed the EMT process, consistent with its established role in driving EMT across multiple tumor types^32^-^34^. The above results suggest that COL1A1 is a key EMT driver gene associated with PCa metastasis.

Collagen is the major extracellular matrix (ECM) component and plays a key role in the tumor microenvironment^35^. COL1A1, the main part of type I collagen, has been linked to carcinogenesis, metastasis, and EMT in many malignancies^36^. In PCa, COL1A1 promotes bone destruction and bone metastasis by disrupting the balance of osteoblasts and osteoclasts^21^. Additionally, increased COL1A1 expression was associated with a poor prognosis in TMPRSS2-ERG negative PCa^20^. However, few studies have shown the relationship between COL1A1 and CTCs in PCa.

Collagen enrichment is one of the hallmarks of malignant tumors, and collagen interacts bidirectionally with cancers^37^. On one hand, tumors regulate the secretion of COL1A1. Cancer-associated fibroblasts (CAFs), mainly regulated by TGF-β, are the primary producers of COL1A1^38^, ^39^. It has been reported that enzalutamide exposure and FOXA1 loss can promote the activation of TGF-β in PCa^40^, ^41^. Additionally, PTEN loss, one of the most frequent genomic aberrations in PCa, can activate CAFs and increase the production of COL1A1^42^, ^43^. These could explain our study's elevated levels of COL1A1 expression in HRPC. On the other hand, COL1A1 can also have an impact on the development of tumors. Collagen fibers can directly mediate the intravasation of tumor cells, promoting the entry of tumors from the primary foci into adjacent tissues and the vascular system^44^; they can also activate AKT/PI3K, MAPK, and ERK1/2 signaling pathways to promote tumor migration^45^, ^46^, as well as act in concert with MMP to enhance invasion^47^. The results of our functional experiments are consistent with the findings of these studies.

This study found a correlation between the NE^+^ CTCs percentage and COL1A1 expression in PCa tissues. Since EMT promotes CTCs formation and COL1A1 is a critical gene for EMT^48^, COL1A1 may account for the high percentage of NE^+^ CTCs in HRPC patients. While the direct interplay between COL1A1 and CTCs remains unexplored in PCa, previous studies in breast cancer models have shown that COL1A1-accumulating mice have larger lung metastases and more CTCs^46^. Additionally, COL1A1 expression was significantly elevated in CTCs compared to primary tumor lesions, and further enriched in CTC clusters relative to single CTCs^49^. This upregulation implies that COL1A1 may not only drive tumor cell dissemination into the bloodstream but also promote the clustering of single CTCs into CTCs clusters with enhanced metastatic potential.

The main limitations of our study are the relatively small number of patients, short follow-up time, and retrospective design. More studies including a large cohort of PCa patients are warranted.

## Conclusion

Our findings identified NE^+^ CTCs ≥ 45% as an independent risk factor for postoperative progression in HRPC patients after RP. Besides, elevated COL1A1 expression was significantly associated with increased NE^+^ CTCs percentages and found to promote EMT in PCa. These results collectively suggest that COL1A1 may drive the emergence of NE^+^ CTCs through EMT activation, positioning it as a potential therapeutic target to inhibit PCa metastasis.

## Supplementary Material

Supplementary methods, figure and tables.

## Figures and Tables

**Table 1:** Clinical characteristics of 208 patients with high-risk prostate cancer.

Variables	Total n (%)	NE^+^ CTCs percentage				Total CTCs count		
		Positive n (%)	Negative n (%)	*P*		Positive n (%)	Negative n (%)	*P*
All cases	208 (100)	168 (80.8)	40 (19.2)			111 (53.4)	97 (46.6)	
Age (y)				0.959				0.276
< 60	41 (19.7)	33 (19.6)	8 (20.0)			25 (22.5)	16 (16.5)	
≥ 60	167 (80.3)	135 (80.4)	32 (80.0)			86 (77.5)	81 (83.5)	
PSA (ng/ml)				< 0.001*				0.008*
< 20	53 (25.5)	33 (19.6)	20 (50.0)			20 (18.0)	33 (34.0)	
≥ 20	155 (74.5)	135 (80.4)	20 (50.0)			91 (82.0)	64 (66.0)	
Biopsy GS				0.234				0.009*
< 8	102 (49.0)	79 (47.0)	23 (57.5)			45 (40.5)	57 (58.8)	
≥ 8	106 (51.0)	89 (53.0)	17 (42.5)			66 (59.5)	40 (41.2)	
cT stage				0.762				0.172
< cT2c	84 (40.4)	67 (39.1)	17 (42.5)			40 (36.0)	44 (45.4)	
≥ cT2c	124 (59.6)	101 (60.1)	23 (57.5)			71 (64.0)	53 (54.6)	
cN stage				0.016*				0.005*
cN0	150 (72.1)	115 (68.5)	35 (87.5)			71 (64.0)	79 (81.4)	
cN1	58 (27.9)	53 (31.5)	5 (12.5)			40 (36.0)	18 (18.6)	
pGS				0.188				0.003*
< 8	95 (45.7)	73 (43.5)	22 (55.0)			40 (36.0)	55 (56.7)	
≥ 8	113 (54.3)	95 (56.5)	18 (45.0)			71 (64.0)	42 (43.3)	
pT stage				0.002*				0.158
< pT3	90 (43.3)	64 (38.1)	26 (65.0)			43 (38.7)	47 (48.5)	
≥ pT3	118 (56.7)	104 (61.9)	14 (35.0)			68 (61.3)	50 (51.5)	
pN stage				0.002*				0.002*
pN0	138 (66.3)	103 (61.3)	35 (87.5)			63 (56.8)	75 (77.3)	
pN1	70 (33.7)	65 (38.7)	5 (12.5)			48 (43.2)	22 (27.7)	
Surgical margins				0.016*				0.040*
Positive	38 (18.3)	36 (21.4)	2 (5.0)			26 (23.4)	12 (12.4)	
Negative	170 (81.7)	132 (78.6)	38 (95.0)			85 (76.6)	85 (87.6)	

Abbreviation: PSA, prostate-specific antigen; biopsy GS, biopsy Gleason score; cT stage, clinical tumor stage; cN stage, clinical lymph node stage; pGS, pathological Gleason score; pT stage, pathological tumor stage; pN stage, pathological lymph node stage. * Significant.

**Table 2:** Univariate and multivariable analysis of predictors for postoperative progression in high-risk prostate cancer patients.

Variable	Univariable analysis			Multivariable analysis	
	HR (95% CI)	P		HR (95% CI)	P
Age (y)	1.05 (0.629-1.766)	0.841		-	-
PSA (ng/ml)	1.49 (0.899-2.470)	0.122		-	-
pGS	3.14 (1.967-5.011)	< 0.001*		1.96 (1.160-3.306)	0.012*
pT stage	3.01 (1.883-4.799)	< 0.001*		1.68 (1.004-2.812)	0.048*
pN stage	3.67 (2.419-5.563)	< 0.001*		2.09 (1.310-3.337)	0.002*
Total CTCs count	1.56 (1.023-2.363)	0.039*		-	-
NE^+^ CTCs percentage	4.60 (2.008-10.536)	< 0.001*		3.31 (1.423-7.702)	0.005*
Surgical margins	2.33 (1.465-3.693)	< 0.001*		-	-

Abbreviation: HR, hazard ratio; CI, confidence interval; PSA, prostate-specific antigen; pGS, pathological Gleason score; pT stage, pathological tumor stage; pN stage, pathological lymph node stage; NE, non-epithelial. * Significant.

## Abbreviations

ADT: androgen deprivation therapy
bGS: biopsy Gleason score
CI: confidence interval
CTCs: circulating tumor cells
cN: clinical lymph node stage
cT: clinical tumor stage
DFS: disease-free survival
DEG: differentially expressed gene
EMT: epithelial-mesenchymal transition
HR: hazard ratio
HRPC: high-risk prostate cancer
IHC: immunohistochemistry
MET: mesenchymal-epithelial transition
NE: non-epithelial
PFS: progression-free survival
PCa: prostate cancer
PSA: prostate-specific antigen
pGS: pathological Gleason score
pN: pathological lymph node stage
pT: pathological tumor stage
RP: radical prostatectomy
t-CTCs: total CTCs
