# Supplementary material for: Non-epithelial Circulating Tumor Cells Enhance Disease Progression in High-risk Prostate Cancer through EMT and COL1A1 Expression
Source: Int J Med Sci. 2025 Feb 28;22(7):1562–73. doi: 10.7150/ijms.107703 (PMC11905264; doi:10.7150/ijms.107703)
Supplement: Supplementary file 1 — Supplementary methods, figure and tables. [file ijmsv22p1562s1.pdf]

Supplementary Materials

Supplementary Methods

Cancer cell lines and cell spiking experiments

Based on expression level and tumor histology, we selected the metastatic (PC3, DU145, LNCaP) and local (22Rv1) PCa cell lines. These 4 PCa cell lines were spiked in 5 ml of blood obtained from healthy donors in a series of dilutions (10, 50, and 100 cells/ 5 mL). The spiked blood samples were processed using the CanPatrol^{TM} platform (SurExam, China). As described previously, the cutoff values were defined as the lowest rates of NE^+ CTCs among the cell lines[1].

CTCs isolation and classification

As described in the prior report, the CanPatrol^{TM} platform was used to isolate CTCs from 5 mL blood in PCa patients[1]. Cell nuclei were stained with 4′,6-diamidino-2-phenylindole, and the leukocytes were identified by CD45 expression using fluorescent microscopy.

We conducted the CTCs classification with the following capture probes, which are specific for epithelial markers EpCAM (R&D, Minneapolis, USA) and CK8/18/19 (R&D, Minneapolis, USA), mesenchymal markers vimentin and twist (BD Bioscience, San Jose, USA), and the leukocyte marker CD45 (Surexam, Guangzhou, China). The non-epithelial (NE^+) CTCs included hybrid and mesenchymal types.

Prior studies have shown the feasibility of detecting EMT-related markers in CTCs via RNA-ISH and the capture probe sequences for the EpCAM, CK8/18/19, vimentin, twist, and CD45 genes [1, 2].

Data source

The PCa dataset from TCGA is comprised of 489 PCa and 51 non-cancerous prostate samples. For the convenience of downstream analysis, all probe identifiers were converted to Ensembl gene IDs using the human genome sequence (GRCh38/hg38)2

and annotation GTF file (GENCODE version 26). Annotation probes were not removed; gene expression analysis was performed only on genes exceeding an average of >1 count per million (CPM). Finally, the genes with read per kilobase million (RPKM) values were utilized for further analysis after filtering.

The HALLMARK_EPITHELIAL_MESENCHYMAL_TRANSITION gene set, contributed by Liberzon A et al., contains 200 EMT-related genes. These genes were related to epithelial-mesenchymal transition, as in wound healing, fibrosis, and metastasis.

The details of datasets from GEO are listed in **Table S1**.

UALCAN and GEPIA2

UALCAN (http://ualcan.path.uab.edu/) is a reliable, comprehensive, and interactive cancer omics data analysis network resource[3]. We used the TCGA analysis module UALCAN and chose the prostate cancer analysis module to investigate the relationship between COL1A1 expression and various Gleason scores across, as well as in different tumor subsets stratified by lymph node stages.

Gene Expression Profiling Interacting Analysis 2 (GEPIA2; http://gepia2.cancer-pku.cn/) is an upgraded version of GEPIA developed by a Peking University project team[4]. Our research used the "Survival Analysis" module of GEPIA2 to generate a Kaplan-Meier curve based on the COL1A1 expression.

Tumor tissue immunohistochemistry (IHC) evaluations

Formalin-fixed, paraffin-embedded tissue specimens were cut into 4 μm sections. Antigen retrieval was conducted in citrate buffer (10 mmol/L, pH 6.0) at 100 °C for 15 minutes, followed by endogenous peroxidase blocking. After primary and secondary antibodies were incubated, sections were treated with 3, 3’-diaminobenzidine (DAB) and counterstained with hematoxylin. Immunostaining was carried out with an antibody for COL1A1. The method for obtaining tissue specimens and immunostaining analysis were conducted as previously described[5]. Briefly, the COL1A1 immunostaining

score was calculated according to the staining intensity and the percentage of positively stained tumor cells. The staining intensity scores ranged from 0 to 3, with 0 for no staining, 1 for weakly stained, 2 for moderately stained, and 3 for strongly stained. The percentage positivity was graded from 0 to 3, with 0 for < 10%, 1 for 10 - 30%, 2 for 31 - 50%, and 3 for > 50%. The total score for COL1A1 expression was calculated as the staining intensity score × the percentage positivity score, which ranged from 0 to 9. COL1A1 expression was classified as "negative" (score 0), "weak" (score 1–4), and "strong" (score 5–9). The staining of each tissue was evaluated by 2 experienced pathologists.

References

1. Wu S, Liu S, Liu Z, Huang J, Pu X, Li J, et al. Classification of Circulating Tumor Cells by Epithelial-Mesenchymal Transition Markers. PLoS One. 2015; 10: e0123976.

2. Cheng B, Tong G, Wu X, Cai W, Li Z, Tong Z, et al. Enumeration And Characterization Of Circulating Tumor Cells And Its Application In Advanced Gastric Cancer. Onco Targets Ther. 2019; 12: 7887-96.

3. Chandrashekar DS, Bashel B, Balasubramanya SAH, Creighton CJ, Ponce-Rodriguez I, Chakravarthi B, et al. UALCAN: A Portal for Facilitating Tumor Subgroup Gene Expression and Survival Analyses. Neoplasia. 2017; 19: 649-58.

4. Tang Z, Kang B, Li C, Chen T, Zhang Z. GEPIA2: an enhanced web server for large-scale expression profiling and interactive analysis. Nucleic Acids Res. 2019; 47: W556-w60.

5. Gu Y, Wang Q, Guo K, Qin W, Liao W, Wang S, et al. TUSC3 promotes colorectal cancer progression and epithelial-mesenchymal transition (EMT) through WNT/β-catenin and MAPK signalling. J Pathol. 2016; 239: 60-71.

Table S1. Information of GEO datasets in this study.

Dataset	Contributors	Platforms	Overall design
GSE60329	Chiorino et al.	GPL14550 Agilent-028004 SurePrint G3 Human GE 8x60K Microarray	54 PCa samples versus 14 normal prostate samples
GSE32269	Cai et al.	GPL96 [HG-U133A] Affymetrix Human Genome U133A Array	22 primary Pca (hormone-dependent) versus 29 metastatic Pca (CRPC)
GSE38241	Aryee et al.	GPL4133 Agilent-014850 Whole Human Genome Microarray 4x44K G4112F	18 multiple anatomically distinct metastases versus 21 normal prostate samples

Table S2. Primer sequences used for qRT-PCR in this study.

Gene Name	Forward Primer	Reverse Primer
COL1A1	5'- GATGGATTCCAGTTCGAGTATG -3'	5'- TGTTCTTGCAGTGGTAGGTGATG -3'
GAPDH	5'- GGAGCGAGATCCCTCCAAAAT -3'	5'- GGCTGTTGTCATACTTCTCATGG -3'

Table S3. The oligonucleotides used in this study.

Gene Name	Target Sequence
si- COL1A1-1	5'-TTG GTG TTG TGC GAT GAC GTG-3'
si- COL1A1-2	5'-CCA UCA AAG UCU UCU GCA ATT-3'

Table S4. Univariate and multivariable analysis for postoperative progression in high-risk prostate cancer patients (COL1A1 included).

Variable	Univariable analysis		Multivariable analysis	
	HR (95% CI)	*P*	HR (95% CI)	*P*
Age (y)	1.05 (0.629–1.766)	0.841	-	-
PSA (ng/ml)	1.49 (0.899–2.470)	0.122	-	-
pGS	3.14 (1.967–5.011)	**< 0.001***	1.95 (1.165–3.253)	**0.011***
pT stage	3.01 (1.883–4.799)	**< 0.001***	1.93 (1.155–3.216)	**0.012***
pN stage	3.67 (2.419–5.563)	**< 0.001***	2.04 (1.300–3.210)	**0.002***
Total CTCs count	1.56 (1.023–2.363)	**0.039***	-	-
NE^+ CTCs percentage	4.60 (2.008–10.536)	**< 0.001***	2.62 (1.119–6.138)	**0.027***
Surgical margins	2.33 (1.465–3.693)	**< 0.001***	-	-
COL1A1	2.48 (1.608–3.812)	**< 0.001***	2.17 (1.398–3.377)	**0.001***

Abbreviation: HR, hazard ratio; CI, confidence interval; PSA, prostate-specific antigen; pGS, pathological Gleason score; pT stage, pathological tumor stage; pN stage, pathological lymph node stage; NE, non-epithelial.

* Significant.

Figure S1. Bioinformatic analysis of epithelial-mesenchymal transition (EMT)-related genes in prostate cancer (PCa). A-D) Volcano plots of differentially expressed genes (DEGs) in TCGA-PRAD, GSE32269, GSE38241, and GSE60329 datasets. **E)** Overall survival (OS) by COL1A1 expression. Data are represented as mean ± standard deviation (SD). The *P*-value was estimated by Student's *t*-test.

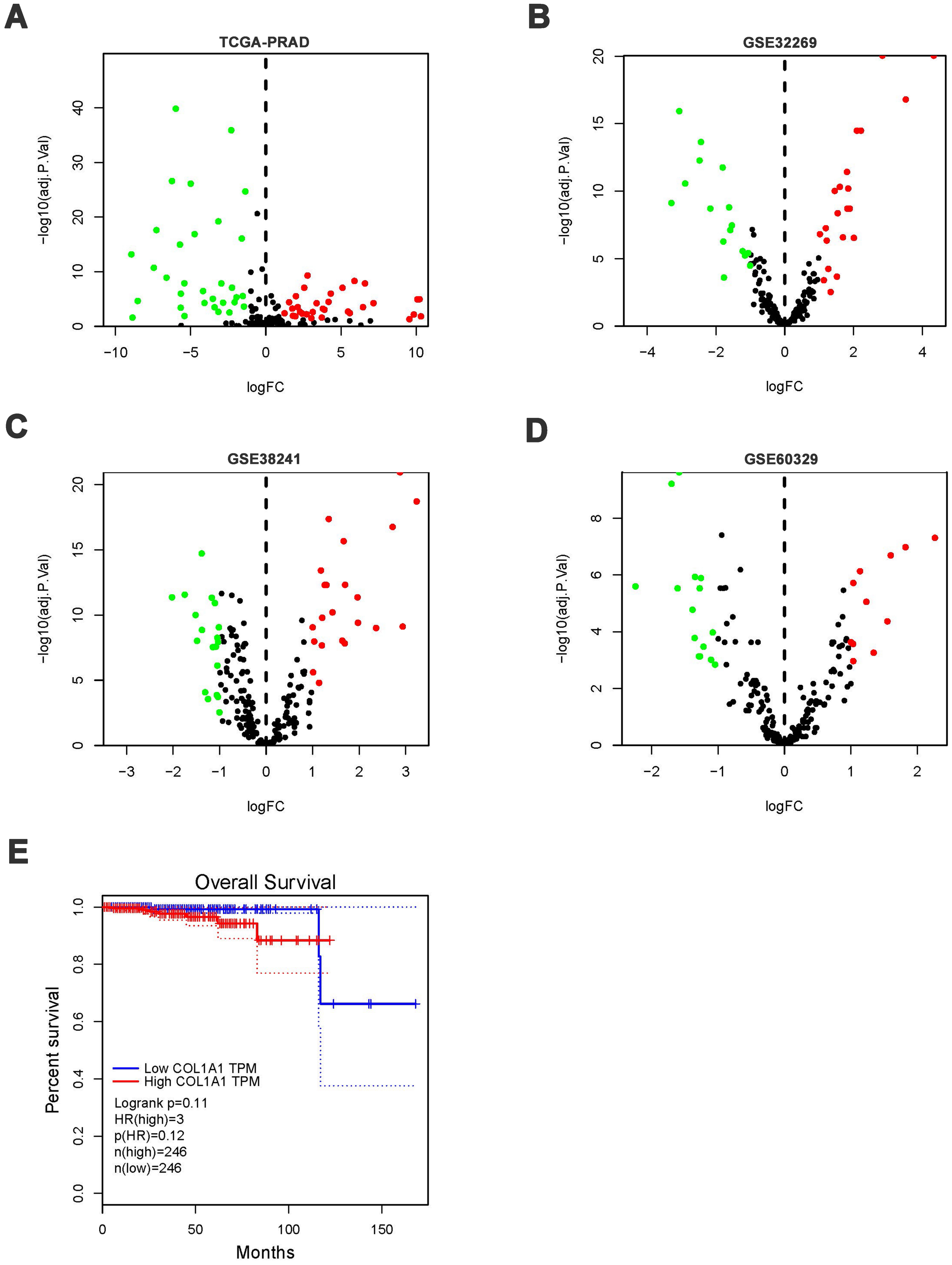
A
TCGA-PRAD
-log10(adj.P.Val)
logFC
B
GSE32269
-log10(adj.P.Val)
logFC
C
GSE38241
-log10(adj.P.Val)
logFC
D
GSE60329
-log10(adj.P.Val)
logFC
E
Overall Survival
Percent survival
Months
Low COL1A1 TPM
High COL1A1 TPM
Logrank p=0.11
HR(high)=3
p(HR)=0.12
n(high)=246
n(low)=246
